# Laparoendoscopic Single-Site Surgery (LESS): A Shift in Gynecological Minimally Invasive Surgery

**DOI:** 10.7759/cureus.32205

**Published:** 2022-12-05

**Authors:** Anisha Ranjan, Ketav S Joshi, Sandhya Pajai, Shazia Mohammad

**Affiliations:** 1 Obstetrics and Gynaecology, Jawaharlal Nehru Medical College, Datta Meghe Institute of Medical Sciences, Wardha, IND; 2 Obstetrics and Gynaecology, Acharya Vinoba Bhave Rural Hospital, Wardha, IND

**Keywords:** sils, single port access, scarless surgery, minimally invasive surgery, single site surgery, single port, single incision, laparoendoscopic single-site surgery

## Abstract

Laparotomy was once the preferred modality of treatment for various gynecological conditions. However, over the years, with the advancements worldwide, a new technique for surgery, laparoscopy, came into play. Since then, laparoscopy is preferred over laparotomy for diagnostic and therapeutic purposes since it was less invasive than laparotomy. Further advancements include laparoendoscopic single-site surgery (LESS), which is a procedure that, as the name implies, only uses one port. It includes using a single incision near the umbilicus in contrast to laparoscopy, which traditionally includes one main port incision and various other side ports. Through the port, multiple devices can be inserted into the cavity. The use of a single port can reduce post-operative complications and help reduce the duration of hospital stays. A single incision near the umbilicus would not leave a very significant scar, and the wound healing time would be comparatively less, reducing the hospital stay time. This novel technique is, therefore, an amalgamation of traditional surgery and recently surfacing minimally invasive surgery. Other modalities which are being used widely include vaginal natural orifice transluminal endoscopic surgery (vNOTES). Since the ports formed are frequently inconspicuous, these procedures leave patients with "scarless" results.

## Introduction and background

Minimally invasive surgery has become an essential part of general surgery. Its development has been one of the most impressive in surgery. Dimitri Ott, Georg Kelling, and Hans Christian Jacobeus were the ones who introduced laparoscopy at the start of the century [1]. It has become a routinely performed procedure in Obstetrics and Gynecology. It can be used as a diagnostic as well as a therapeutic tool. Technological advances led to the development of better-quality, high-resolution cameras, video cams, and much safer instruments allowing surgeons to use laser and electrical energy, including harmonic scalpels, for cutting and cauterizing tissues or achieving homeostasis. These developments make the surgeon's job considerably easier with high-quality visualization while shortening hospital stays for patients.

Laparoscopy has also decreased adhesion formation, the incidence of infections, and other laparotomy-related problems, which has attracted surgeons to this technique. Since every surgical incision has a danger of bleeding, infection, organ damage, and the development of hernias, researchers have concentrated on decreasing the number of surgical ports. The development of improved devices appropriate for even less invasive surgery makes it feasible to do the same procedure with fewer incisions [2]. Continued efforts are being made to refine the technique of laparoscopy. Also, considering the cosmetic factor associated, a more feasible method known as laparoendoscopic single-site surgery (LESS) has come into the picture [3].

In contrast to laparoscopy, where multiple ports are created in addition to the main port, LESS involves the formation of only a single port near the umbilicus. This single port can act as a portal through which multiple instruments can be inserted into the patient's abdominal cavity. Therefore, various gynecological procedures which are not very invasive can be done. Not only in the field of gynecology, but it has also shown promising results in various other procedures like cholecystectomy, nephrectomy, hemicolectomy, and appendicectomy [4,5].

This review was conducted to determine other potential benefits and drawbacks, primarily in terms of intra-operative and post-operative complications, as well as to evaluate and validate the feasibility and safety of LESS in patients with the gynecological disease compared to traditional laparoscopy and vaginal natural orifice transluminal endoscopic surgery (vNOTES).

## Review

Role of LESS in various gynecological procedures

Uterine fibroids, which are commonly seen in women of reproductive age, can be removed with the use of less invasive techniques, which include LESS. The prevalence of fibroids is as high as 77% among women of the reproductive age group. Hence less invasive surgeries like LESS become a safer option. Myomectomy can be easily performed to resect these leiomyomas in the early stages [6]. Recent clinical studies mentioned in Table 1 have shown that laparoendoscopic single-site myomectomy (LESS-M) is much more feasible and safe [7].

Regarding procedures like hysterectomy, various minimally invasive procedures like laparoscopy, vNOTES, and LESS can be done, as depicted in Table 1. Previously, a hysterectomy was performed by laparotomy, which resulted in side effects such as severe blood loss and repeated blood transfusions [8]. Therefore, newer techniques were developed to reduce these complications. Studies were carried out to compare the efficacy of vNOTES and LESS. There was not much of a distinction between the two. The only thing that differentiated the two was that the post-operative vaginal pain seen in patients who underwent LESS was relatively less than in those who underwent vNOTES (Tables 1, 3, 4) [9]. Another factor taken into account, which is also mentioned in Tables 1, 3, was the fact that LESS took more time than vNOTES [10,11].

Table 1 consists of various variables taken into account in order to compare LESS with other modalities like vNOTES and conventional laparoscopy.

**Table 1:** Comparison between LESS and other modalities like vNOTES and conventional laparoscopic surgery (CLS)

Name of the article	Comparison between variables that were taken into account while comparing various modalities of gynaecological surgeries
Noh et al., 2022, Korea [12].	In this study, some of the patients underwent conventional laparoscopic surgery (CLS) while others underwent vNOTES hysterectomy. It was noticed that the operative time of CLS laparoscopic operation was longer and there was a longer port-installation time in patients of vNOTES.
Park et al., 2021, Korea [9].	LESS required a significantly higher operative time, and more post-operative vaginal pain was observed in the patients who underwent vNOTES.
Chen et al., 2020, China [13].	The intestinal function was restored earlier in patients who underwent vNOTES. Shorter hospital stays in case of vNOTES and indwelling time of catheter was much less in vNOTES.
Zhou et al., 2021, China [6].	CLS has a shorter operative time when compared to laparoendoscopic single-site myomectomy (LESS-M). Blood loss was more in the case of LESS-M when the fibroid size was more than 8 cm, and patients who underwent LESS-M had higher scar satisfaction scores.
Pontis et al., 2016, Israel [14].	A more frequent conversion rate was seen in patients who underwent LESS when compared to those who underwent CLS.

Table 2 shows the outcomes of interest that were compared.

**Table 2:** Criteria for comparison

Intra-operative	Operative time, blood loss, extra port formation
Post-operative	Post-operative vaginal pain, hospital stay, cosmesis

Various complications like blood loss, hemoglobin drop, conversion to laparotomy, or extra port formation were considered, and a comparison was made between different modalities, as depicted in Table 3. In their study, Park et al. found that vNOTES (vaginal natural orifice transluminal endoscopic surgery) required significantly less operative time when compared to LESS in patients undergoing hysterectomy [9]. A shorter operative time in vNOTES can be attributed to the fact that it does not require opening and closing procedures at the umbilicus [9]. Noh et al. found that vNOTES was faster and less time-consuming than conventional laparoscopic surgery (CLS). However, the port-installation time was longer in the case of vNOTES [12]. Longer operative time was also seen in patients undergoing myomectomy by LESS compared to conventional laparoscopy, as stated by Zhou et al. In his study, he also learned that blood loss was more in the case of LESS-M when the fibroid size was more than 8 cm [6]. Zhou et al. also noted that conventional laparoscopic myomectomy (CLM) takes less time than LESS-M when a fibroid's diameter is less than 8 cm, it is placed in the posterior wall, or there are less than four fibroids.

**Table 3:** Intra-operative complications

Intraoperative Complications		
Complications	Study	Inference
Operative time	Park et al., 2021, Korea [9]. Noh et al., 2022, Korea [12]. Zhou et al., 2021, China [6].	LESS required a significantly higher operative time than vNOTES. conventional laparoscopic surgery has a longer operative time compared to vNOTES. LESS-M has a longer operative time when compared to CLM.
Blood loss	Zhou et al., 2021, China [6].	Blood loss is more in the case of LESS-M when the fibroid size is more than 8 cm.
Extra port	Pontis et al., 2016, Israel [14].	A more frequent conversion rate was seen is LESS when compared to MLS.

Table 4 compares various post-operative complications which were documented post-surgery. Compared to LESS, post-operative complications like post-operative vaginal pain were more in the case of vNOTES [9]. The pain was calculated using the VAS (visual analog score) scoring system. The pain was measured at 16 and 24hrs. More pain in vNOTES could be accounted to vaginal sutures placed during the surgery. Compared to individuals who received conventional laparoscopic surgery, those who underwent LESS had much lower rates of scar development [6]. The length of hospital stay for patients who underwent vNOTES and those who underwent LESS were compared by Chen et al. Patients who had vNOTES had noticeably shorter hospital stays [13,15].

**Table 4:** Post-operative complications

Post-Operative Complications		
Complications	Study	Infernece
Post-operative vaginal pain	Park et al., 2021, Korea [9].	Less post-operative vaginal pain was observed in the patients who underwent LESS.
Hospital stay	Chen et al., 2020, China [13].	More extended hospital stays in case of LESS.
Cosmesis	Zhou et al., 2021, China [6].	LESS-M has significantly higher scar satisfaction scores compared to CLM.

After reviewing multiple articles, we tried to compare various available surgical modalities, including LESS, vNOTES, and conventional laparoscopy surgery, as mentioned in Tables 1-4. They were compared based on various intra-operative and post-operative parameters, including blood loss, operative time, extra port formation and cosmesis, hospital stay, and post-operative vaginal pain (Table 2).

LESS is a rapidly developing entity in the surgical field, especially for gynecological operations. A significant number of surgeons are adopting this method. For numerous surgeries, it has been reported to be effective and safe. Hysterectomy, myomectomy, and various other benign and malignant conditions can be operated on using this technique. Ectopic pregnancy, myomectomy, and hysterectomy for both benign and malignant lesions have all been shown to be feasible and safe using LESS [16-18].

LESS had a higher conversion rate. As LESS requires the formation of only a single port, crowding of instruments can occur, accounting for higher conversion rates [19,20]. vNOTES take less time to perform than LESS in terms of operating time. This is explained by the fact that fewer incisions and sutures are required. Although various research suggested that one advantage of vNOTES was improved pelvic structure visualization, vNOTES also carry the risk of damaging the urinary tract during surgery [21,22]. However, due to increased incisions, traditional laparoscopy takes much longer to execute. Obesity-related studies have demonstrated that hysterectomy is possible in obese people. However, other authors believe that the LESS approach is ineffective in obese patients due to the increased distance between the surgical site and vital organs caused by going in via the umbilicus [23-25]. If the patient has abdominal mesh from previous umbilical hernia surgery, it is a strict contraindication for LESS [26].

In terms of blood loss, in their study, Zhou et al. considered the amount of blood loss that took place intra-operatively during myomectomy. When the diameter of the fibroid was less than 8 cm, or when the fibroid was located on the fundus, lateral wall, or anterior wall, and when the fibroid count was less than four, in such cases there was no statistical significance between the amount of blood loss in patients who underwent LESS from those who underwent CLM. However, when the size of the fibroid was more than 8 cm, LESS had more blood loss than CLM. Furthermore, the operation time of the CLM is shorter than that of the LESS-M when the diameter of a fibroid is less than 8 cm, a fibroid is placed in the posterior wall, or there are fewer than four fibroids [6]. The shorter time could be because more space was available for surgery in the case of large fibroids.

As for the pain experienced post-operatively, vNOTES patients had more vaginal pain than LESS patients [9]. When the pain was calculated using the VAS score after 16 and 24 hours, more vaginal pain was seen post-operatively in patients who underwent vNOTES [9]. Huang et al. found out that vNOTES VAS score 24 hours post-operatively was significantly less compared to transumbilical-LESS (TU-LESS) group [27]. According to Yang et al., there was no discernible difference between the vNOTES and TU-LESS hysterectomy VAS scores at 12 and 24 hours following surgery [28]. Whether vNOTES is superior to TU-LESS is still not very clear.

Nowadays, it is essential to take cosmesis into mind. LESS-M has significantly higher scar satisfaction scores compared to CLM. According to two prospective studies, the cosmetic score of single-site laparoscopic hysterectomy was greater than that of a typical laparoscopy procedure, proving the procedure's superior cosmetic benefits [29,30]. Therefore, the satisfaction level of incision in LESS patients is higher than the conventional laparoscopy. For the treatment of large ovarian cysts, LESS surgery with an angiocatheter needle, leaving just a little post-operative scar, was considered possible [31]. There is a propensity to choose conventional laparoscopy surgery (CLS) over LESS in an emergency as operative time is much faster, thereby preventing complications [7].

Interestingly, the LESS and CLS groups had comparable hospital stays and total morbidity [14]. Whereas more extended hospital stays in the case of LESS were noted compared to vNOTES [13]. Therefore single-site surgery can be performed faster, and the patient can be sent home as soon as possible. Additionally, various health systems may impact a patient's hospital stay and discharge [32].

Further research is necessary to find out the actual cost and its relation to various other factors which may influence the final cost of the surgery and the outcome of the surgery.

Last but not least, it can be said that LESS, traditional laparoscopy, and vNOTES are equivalent modalities based on all the above characteristics. Each patient must get individualized care depending on the requirements, potential complications, and whether the operation is elective or an emergency.

## Conclusions

After reviewing various articles, it can be concluded that LESS does not significantly differ from conventional laparoscopic surgery (CLS) in terms of the management of gynecologic diseases. For some individuals, LESS may be suggested as an alternative to CLS and vNOTES since it is a feasible and reliable technique. It has lower postoperative pain scores and better scar satisfaction scores. A single incision around the umbilicus would not leave an unsightly scar, and the wound healing period would be shorter, minimizing the length of hospital stay. Each patient must get tailored treatment depending on the needs, potential problems, and whether the procedure is elective or an emergency. As this review article is insufficient, additional research is needed to learn more about the efficacy of LESS and its advantages over various other modalities available.
